# Supplementary material for: New Self-Healing Metallosupramolecular Copolymers with a Complex of Cobalt Acrylate and 4′-Phenyl-2,2′:6′,2″-terpyridine
Source: Polymers (Basel). 2023 Mar 16;15(6):1472. doi: 10.3390/polym15061472 (PMC10053782; doi:10.3390/polym15061472)
Supplement: Supplementary file 1 [file polymers-15-01472-s001.zip › polymers-2213486-supplementary.pdf]

polymers

MDPI

Article

New Self-Healing Metallosupramolecular Copolymers with a Complex of Cobalt Acrylate and 4′-Phenyl-2,2′:6′,2″-terpyridine

Evgeny S. Sorin [1,2], Rose K. Baimuratova [1], Igor E. Uflyand [3,4], Evgeniya O. Perepelitsina [1], Denis V. Anokhin [1,2], Dmitry A. Ivanov [1,2,5] and Gulzhian I. Dzhardimalieva [1,3,*]

[1] Federal Research Centre of Problems of Chemical Physics and Medicinal Chemistry RAS, 142432 Chernogolovka, Russia; sorin_es@mail.ru (E.S.S.); rozab@icp.ac.ru (R.K.B.); jane@icp.ac.ru (E.O.P.); anokhin@icp.ac.ru (D.V.A.); dimitri.ivanov.2014@gmail.com (D.A.I.)

[2] Department of Fundamental Physical and Chemical Engineering, Lomonosov Moscow State University, 119234 Moscow, Russia

[3] Moscow Aviation Institute, National Research University, 125993 Moscow, Russia; ieuflyand@sfedu.ru

[4] Department of Chemistry, Southern Federal University, 344090 Rostov-on-Don, Russia

[5] Institut de Sciences des Matériaux de Mulhouse (CNRS UMR 7361), 68057 Mulhouse, France

* Correspondence: dzhardim@icp.ac.ru; Tel.: +7-(496)-522-7763

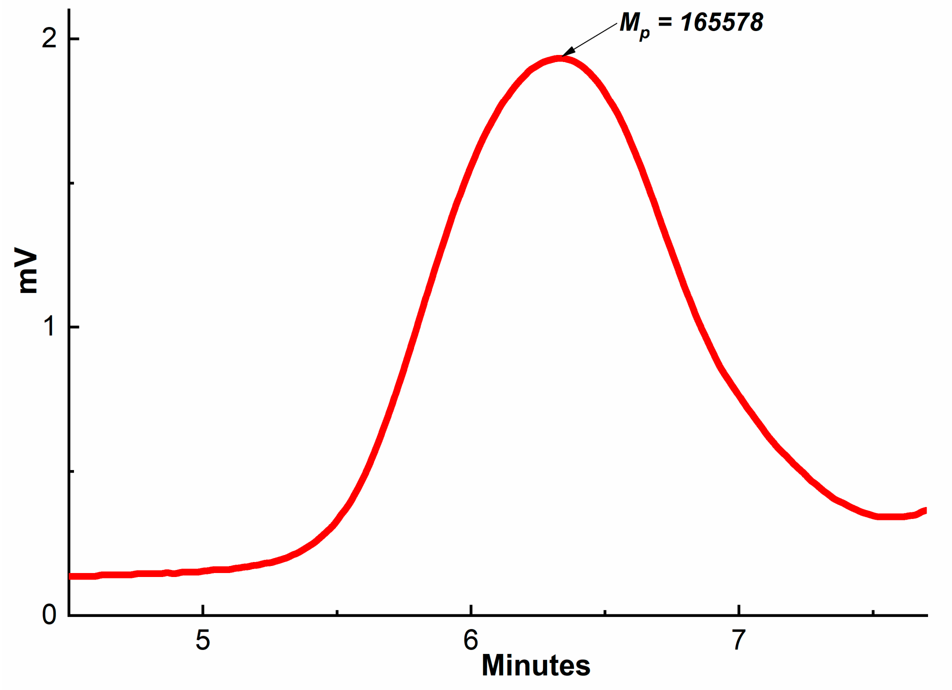

Figure S1. Chromatogram of the ash part of Copolymer 3.

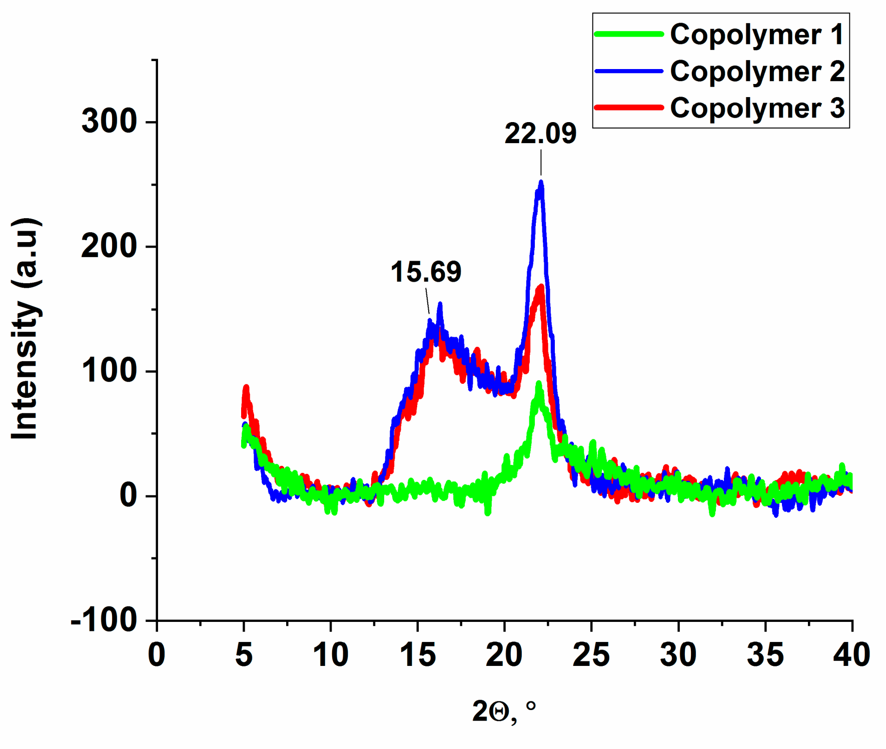

Figure S2. XRD patterns of copolymers with 1wt.% $CoAcr_2PhTpy$: Copolymer 1 – AAm/AAc = 84.5/14.5; Copolymer 2 - AAm/AAc = 49.5/49.5; Copolymer 3 - AAm/AAc = 14.5/84.5.

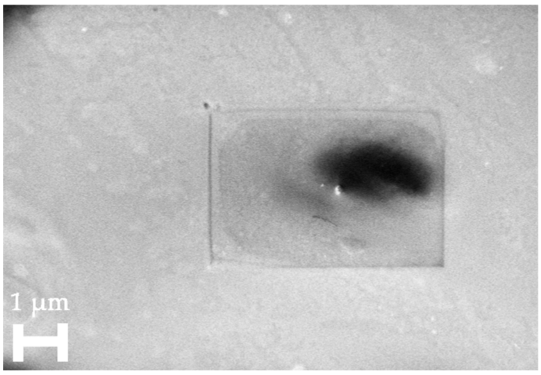

(a)

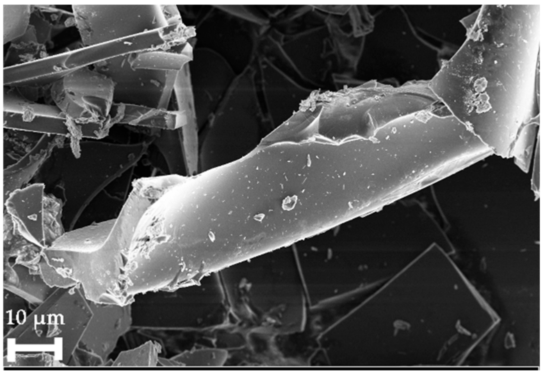

(b)

Figure S3. SEM photos of the original Copolymer 1 (a) and the thermolysis product obtained at 600 °C in a self-generating atmosphere (b).
